# Perception of Solar Eclipses Captured by Art Explains How Imaging Misrepresented the Source of the Solar Wind

**DOI:** 10.1177/2041669515613710

**Published:** 2015-11-26

**Authors:** Richard Woo

**Affiliations:** Jet Propulsion Laboratory, California Institute of Technology, CA, USA

**Keywords:** Solar eclipse, perception, art, imaging, solar corona, solar wind

## Abstract

The visible corona revealed by the natural phenomenon of solar eclipses has been studied for 150 years. A turning point has been the discovery that the true spatial distribution of coronal brightness can neither be seen nor imaged on account of its unprecedented dynamic range. Howard Russell Butler (1856–1934), the painter of solar eclipses in the early 20th century, possessed the extraordinary skill of painting from memory what he saw for only a brief time. His remarkable but forgotten eclipse paintings are, therefore, ideal for capturing and representing best the perceptual experience of the visible corona. Explained here is how by bridging the eras of visual (late 19th century) and imaging investigations (since the latter half of the 20th century), Butler’s paintings reveal why white-light images misled researching and understanding the Sun’s atmosphere, the solar wind. The closure in understanding solar eclipses through the convergence of perception, art, imaging, science and the history of science promises to enrich the experience of viewing and photographing the first solar eclipse of the 21st century in the United States on 21st August 2017.

Shown in Figure 1 (top) is an image of the 1966 solar eclipse taken with the Newkirk camera of the High Altitude Observatory (Newkirk, Dupree, & Schmahl, 1970). Equipped with a filter that removed an average radial falloff in brightness, it revealed a corona dominated by narrowing features known as coronal streamers. Sinclair (2012) has written an excellent summary of his rediscovery of Butler’s paintings of the 1918, 1923, 1925, and 1932 solar eclipses. The painting of the 1925 eclipse is shown in Figure 1 (bottom). Although it precedes the image in time by four decades, the similarity of the streamers is inescapable, and reinforces that visual perception responds to the high dynamic range of brightness by likewise removing an average radial falloff in brightness (Woo, 2011).

**Figure 1.**
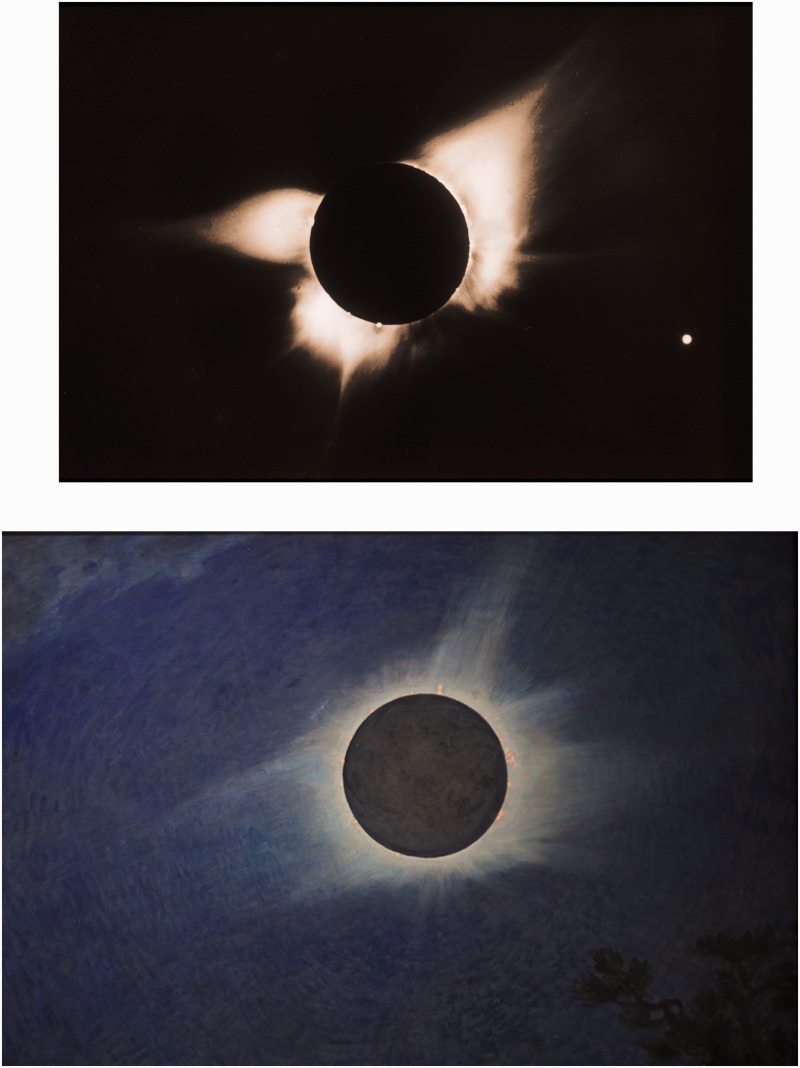
Top: Image of the 1966 eclipse, courtesy of the High Altitude Observatory (HAO), University Corporation for Atmospheric Research (UCAR), Boulder, Colorado sponsored by the National Science Foundation. Bottom: Butler’s painting of the 1925 eclipse, kindly provided by the Princeton University Art Museum.

That Butler’s paintings capture best the perceptual experience of the visible corona is not surprising, given that he studied physics and graduated before becoming a successful landscape and portrait artist, the most well known of his portraits being those of Andrew Carnegie. Although he had never seen a total eclipse, he drew up meticulous scientific plans to prepare for the 1918 eclipse (Butler, 1919), and executed them with the same artistic ability that served him well in painting transient phenomena such as sunsets and the aurora borealis. The finished painting eventually graced the Hayden Planetarium’s rotunda (Lawrence & Milner, 2000). Astronomers who saw the painting and the 1918 eclipse looked upon it as a marvel of perfection, true both to form and colour, a work of art that had the advantage of being scientifically accurate (Mitchell, 1919).

Streaming charged particles from the Sun are known as the solar wind. Scientists studying the solar wind turned to eclipse images for clues to its source at the Sun. One difference sets Butler’s paintings apart from the imaged corona. As seen in Figures 1 and 2, dark regions at the base of the corona near the poles of the Sun give the impression of holes opening up and diverging from the limb of the Sun. They are present in the imaged corona (top), but not in the paintings (bottom). Diverging-hole regions are crucial for exploring and understanding the solar wind, because they identified the source of the solar wind (Hundhausen, 1977; Zirker, 1977). The recent Ulysses mission, the first to send a spacecraft out of the ecliptic plane, measured the high-latitude fast wind and used coronal images to confirm diverging polar coronal holes as its source at the Sun (Balogh, Marsden, & Smith, 2001).

**Figure 2.**
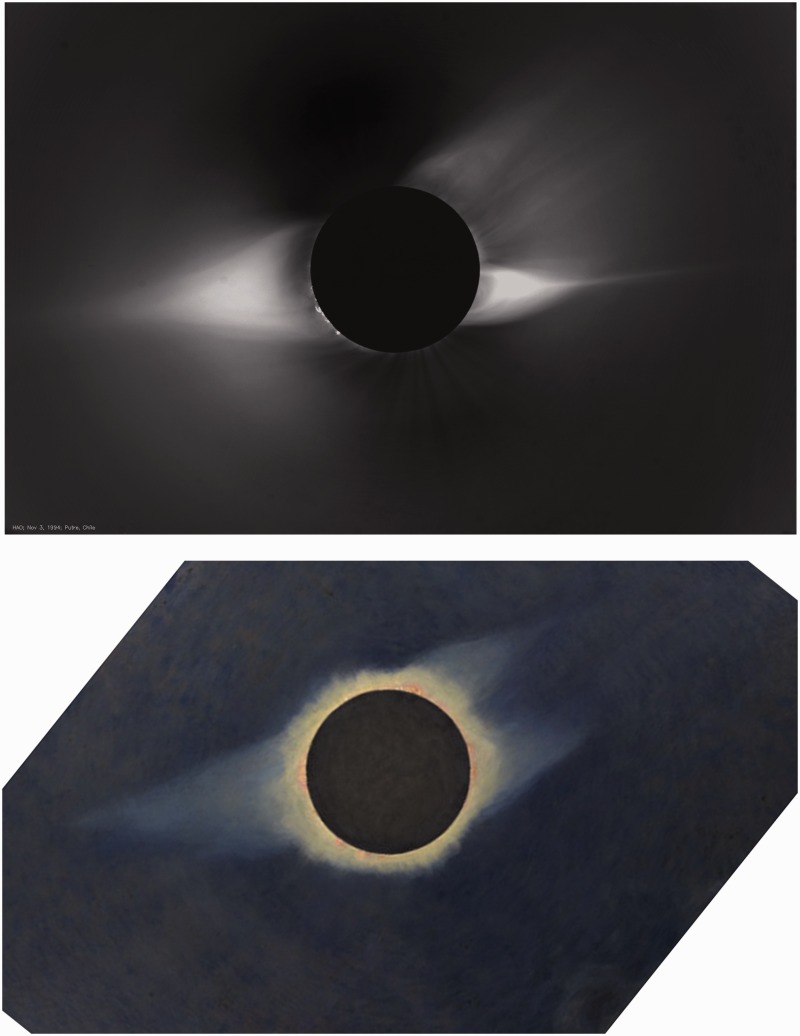
Similar to Figure 1, except image is of the 1994 eclipse, courtesy of the High Altitude Observatory (HAO), University Corporation for Atmospheric Research (UCAR), Boulder, Colorado sponsored by the National Science Foundation, and Butler’s painting is of the 1932 eclipse, kindly provided by the Princeton University Art Museum.

Figure 3, a reproduction of Figure 4 in Woo (2005), shows a coronal image obtained by the ground-based High Altitude Observatory Mauna Loa Mk III K–coronameter (Fisher, Lee, MacQueen, & Poland, 1981). It demonstrates how diverging-hole regions appear and disappear in images when the level of brightness intensity is adjusted. When the level of brightness of Figure 3a is increased, the ‘missing’ corona from the diverging-hole regions appears in Figure 3b while the streamers remain. The missing corona from the image in Figure 3a is confirmed by its detection in the underlying brightness measurements. These results illustrate that, consistent with Butler’s paintings, the diverging-hole regions actually represent regions of the corona whose extent, determined by the threshold sensitivity of the instrument or the naked eye, is shortest (Woo, 2005).

**Figure 3.**
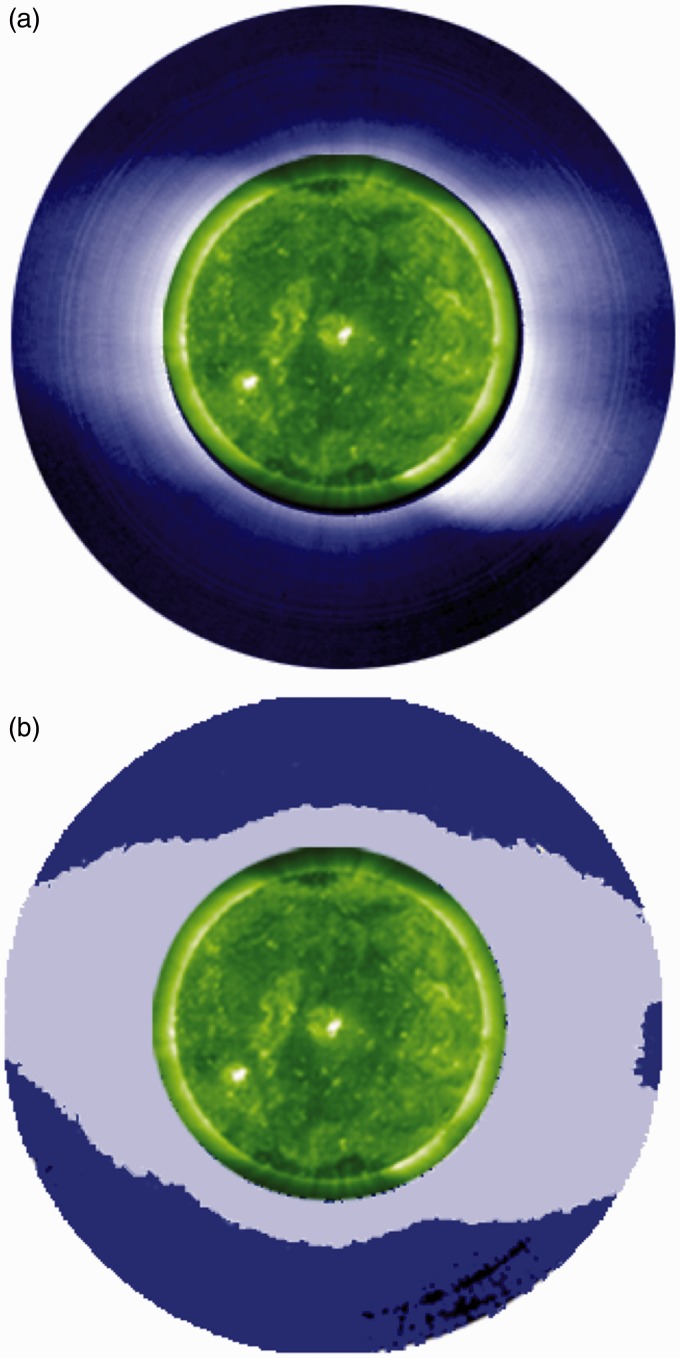
From Woo, R. Solar Physics, 231, 71–85, 2005. (a) White-light image of January 17, 1997 taken by HAO Mauna Loa Mk III K-coronameter and displayed with artificial vignetting to enhance the dynamic range of brightness. (b) Same as (a) except brightness and contrast are increased to reveal the radial extent of the detected corona.

Coronal images informed first on the process of visual perception (Carbon, 2014; Gregory, 1970) through the presence of coronal streamers (Woo, 2011). Visual perception captured in Butler’s paintings now informs on the processing of coronal images through the absence of diverging-hole regions. Unlike visual perception, producers of the images controlled the level of the subtracted radial falloff in brightness without affecting the presence of streamers. Solar wind scientists favoured the level corresponding to diverging-hole regions, because these identified the source of the solar wind. The true brightness distribution can only be represented by quantitative measurements. Investigations based on them confirm the source of fast solar wind is not limited to diverging-hole regions at the Sun (Woo & Habbal, 1999), reinforcing that determining the source of the solar wind requires exploration that goes beyond the use of imaging (Darius, 1990) and our senses (Tyson, 2001).

In conclusion, the trust of coronal imaging in solar wind research reflects the comments by Kemp (2014) in regard to the production and manipulation of images in this digital age:

“In the broadest sense, every act of representation is purposefully selective, just as our acts of seeing. With even the most basic of photographs, depth of field, focus, point of view, lighting, and exposure all serve advertently or inadvertently to include or emphasize some features to exclude or downplay others. This is to say nothing of its subsequent printing and transmission. The scientist, even more than the casual maker of family snapshots, is in the business of selective visual pointing. And the scientist makes sure that the look of the image manifests all the signs of authenticity that are current at the time of its making and reception.

The essential nature of the issue of visual trust in representations has not changed over time, even if the scope for convincing deception has increased in ways that increasingly defy ready detection. We have in practice to work with levels of visual trust, otherwise we could not realistically proceed. Visually, we are trusting beings. As such, our current vulnerability is not essentially different in kind from our predecessors’; it is just far greater in extent.”
